# Duration and compliance with antidepressant treatment in immigrant and native-born populations in Spain: a four year follow-up descriptive study

**DOI:** 10.1186/1471-2458-12-256

**Published:** 2012-05-11

**Authors:** Ins Cruz, Catalina Serna, Montserrat Ru, Jordi Real, Jorge Soler-Gonzalez, Leonardo Galvn

**Affiliations:** 1 Primary Care Research Institute IDIAP J Gol, Catalan Institute of Health, Rambla de Ferran, 44, 3, 25007, Lleida, Spain; 2 Regional Primary Care Management Office, Catalan Institute of Health, University of Lleida, Rambla de Ferran, 44, 3, 25007, Lleida, Spain; 3 University of Lleida, Rambla de Ferran, 44, 3, 25007, Lleida, Spain; 4 Biostatistics Department, University of Lleida, Montserrat Roig, 2, 25006, Lleida, Spain; 5 Pharmacy Unit, Catalan Department of Health, Rovira Roure, 2, 25006, Lleida, Spain

## Abstract

**Background:**

Non-compliance with antidepressant treatment continues to be a complex problem in mental health care. In immigrant populations non-compliance is one of several barriers to adequate management of mental illness; some data suggest greater difficulties in adhering to pharmacological treatment in these groups and an increased risk of therapeutic failure.

The aim of this study is to assess differences in the duration and compliance with antidepressant treatment among immigrants and natives in a Spanish health region.

**Methods:**

Population-based (n=206,603), retrospective cohort study including all subjects prescribed ADT between 2007 and 2009 and recorded in the national pharmacy claims database. Compliance was considered adequate when the duration was longer than 4months and when patients withdrew more than 80% of the packs required.

**Results:**

5334 subjects (8.5% of them being immigrants) initiated ADT. Half of the immigrants abandoned treatment during the second month (median for natives=3months). Of the immigrants who continued, only 29.5% presented good compliance (compared with 38.8% in natives). The estimated risk of abandoning/ending treatment in the immigrant group compared with the native group, adjusted for age and sex, was 1.28 (95%CI 1.16-1.42).

**Conclusions:**

In the region under study, immigrants of all origins present higher percentages of early discontinuation of ADT and lower median treatment durations than the native population. Although this is a complex, multifactor situation, the finding of differences between natives and immigrants in the same region suggests the need to investigate the causes in greater depth and to introduce new strategies and interventions in this population group.

## Background

Non-compliance with prescribed antidepressant treatment (ADT) is one of the main problems in the management of depression. Though it is unanimously recommended that this treatment should be continued after the acute phase to reduce the risk of relapse, the research conducted to date reveals a high percentage of early drop-out and low adherence to treatment [1,2]. In our environment, a recent 5-year follow-up study of 7525 patients found that 56% abandoned treatment during the first four months and that only 22% maintained satisfactory adherence after this period [3].

With the huge increase in worldwide migratory movements in the last decade, the health of the immigrant population has become a matter of growing concern to health authorities. Mental health in particular has become a priority study objective in this population, since it is broadly accepted that migration is a stress factor which may lead to the development of psychiatric illness.

Adequate management of mental illness is made especially difficult in this population by a number of factors: the limited access to psychiatric care, the difficulty of diagnosis due to cultural differences such as low rates of reporting of symptoms, and, in many cases, poor understanding and acceptance of the therapy prescribed. To date, few studies have compared adherence to ADT in natives and immigrants; most of the data available on this issue come from the analysis of factors associated with non-compliance in general.

In Spain, the public health system guarantees access and partial pharmacy coverage for all residents registered in the census, of which immigrants comprise 12.2% (1 January 2010) [4]. Nonetheless, we hypothesized that immigrants would have greater difficulties in adhering to treatment than natives and that the risk of treatment failure would be higher in the more vulnerable groups.

We decided to examine the possible differences between natives and immigrants with regard to the duration and adherence to prescribed ADT.

## Methods

The study was performed in the health region of Lleida (Spain), a region with an active population of 206,603 people between the ages of 15 and 64years according to the central national health insurance registry in 2007. This registry includes all residents who are on the census list of a municipality in the region. In 2007 foreigners represented 13.5% of residents recorded in the census. Most of these foreigners were economic migrants from Eastern Europe, the Maghreb, Latin America, Sub-Saharan Africa and elsewhere.

Study population: population-based retrospective cohort of all subjects aged between 15 and 64years prescribed treatment with antidepressant drugs between 1 January 2007 and 31 December 2009.

The study included all individuals in the region who had received at least one pack of antidepressants in 2007 but who had not received antidepressants in the previous 12months. Subjects who had received ADT with amitriptyline alone were excluded, because of the habitual use of this drug in the treatment of other pathologies such as neuropathic pain or migraine. Individuals who moved to other regions or who died were also excluded from the analysis.

For each individual, age in 2007, sex, country of origin, units of antidepressants from the NO6A subgroup in the ATC classification system [5] dispensed at pharmacies, the active ingredient, and month and year of dispensation were recorded.

The sociodemographic data were obtained from the Primary Care Information System. Data on exposure to drugs were obtained from the regional claims database. In Spain the public health system pays 90% of the price of antidepressant drugs officially prescribed to the population with statutory health insurance. Information on the packs dispensed per person is recorded in an individual database for each health region. We combined the information from these two databases using a common personal identifier; the information was then treated anonymously in the subsequent analysis.

For this study, immigrants were defined as those from low or middle-income countries according to the classification of the World Bank based on per capita GDP [6]. If the country of origin was unknown, only those recorded in the central insurance registry before 1 January 2003, when the percentage of immigrants in the country was below 5%, were considered as natives. Otherwise, subjects whose country of origin was unknown were excluded from the study. The immigrants were grouped according to area of origin into Eastern Europe, Maghreb, Sub-Saharan Africa, Latin America, and Others.

Treatment **duration** was calculated for each patient as the number of months between the first dispensation in 2007 and the last during the study period. If patients did not withdraw medication during a period of six consecutive months this was considered as the end of the episode. Discontinuations of below six months, on the other hand, were included in the duration of the episode. If a patient had presented more than one episode during the study period, only the first one was included. Treatment duration for patients who had not completed treatment by the end of follow-up was considered as censored.

The use of the terms adherence and compliance was discussed by the research team and standardized. In the literature, the terms are often used interchangeably and the definitions used by different authors do not always coincide. Although in general the use of the term adherence is preferred because of its connotation of voluntary cooperation, in this case we define them as follows.

Treatment **adherence** was defined on the basis of the medication possession ratio. For each antidepressant the number of units per month that a patient needed according to the daily dose defined by the WHO Collaborating Centre for Drugs Statistics Methodology was recorded. On this basis, the ratio between the number of units obtained at the pharmacy and the theoretical number of units required according to the duration of the episode was obtained. Ratios of adherence above 80% were considered adequate.

By consensus, **compliance** was considered to be adequate in patients with a treatment duration of more than four months and an adherence of more than 80% until the end of treatment.

The cohorts characteristics with respect to the variables analysed were expressed as frequencies and percentages. The distribution of treatment duration was expressed by the means, medians, 25th and 75th percentiles and 95% confidence intervals (95%CI).

The distribution of treatment duration and the survival function were estimated using the Kaplan-Meier method. The survival curves were compared between groups using the Log-Rank test. To determine which factors were related to the treatment duration and the risk of abandonment/completion, a Cox multivariate model was applied. This model was used to estimate the hazard ratios (HR) and their corresponding 95%CI of ending/abandoning treatment. The same model was used to estimate the adjusted survival curves. The analysis of treatment duration was performed for the entire patient cohort and also for the subsample that completed the first month of treatment. P values below 0.05 were considered statistically significant.

This study was approved by the Ethical Committee of Clinical Research of the IDIAP J Gol (Primary Care Research Institute, Spain).

## Results

### Description of samples

In 2007, 5,334 subjects began treatment with AD, that is, 2.58% of the population of the health region (1.37% of the immigrant population and 2.81% of the natives). Of the patients who began treatment, 91.5% were natives and 8.5% immigrants. The distributions by area of origin and age group are shown in Table 1.

**Table 1:** Distribution of age in the sample according to area of origin.

	Age groups in years (%)					**Total (n)**
	**15-24**	**25-34**	**35-44**	**45-54**	**55-64**	
**NATIVES**	8.2	17.6	24.3	25.5	24.4	4883
**IMMIGRANTS**	12.2	42.1	28.2	11.8	5.8	451
**Latin America**	10.8	35.0	31.2	16.6	6.4	157
**Eastern Europe**	16.4	46.1	24.8	7.9	4.8	165
**Maghreb**	9.8	44.1	26.5	11.8	7.8	102
**Sub-Saharan Africa**	0	60.9	30.4	8.7	0	23
**Others**	25.0	0	75.0	0	0	4

### Duration of AD treatment

Of all the patients who began ADT in the study period, 37.7% (2013) withdrew medication only during the first month of follow-up (48.1% of immigrants compared with 36.8% of natives).

The characteristics of the patients who abandoned treatment during the first month (early discontinuation) were compared with the continuers (Table 2). The percentage of immigrants in the early discontinuation group was higher than in the group that continued beyond this period, with statistically significant differences.

**Table 2:** Characteristics of patients who only received treatment for the first month with regard to sex, age and origin, compared with patients who remained in treatment more than one month

	Treatment duration		**p**
	**1month**	**>1month**	
**N**	2013	3321	
**Male**	35.9%	33.8%	0.116 (2)
**Mean age (years)**	45.5	46.2	0.064 (1)
**IMMIGRANTS**	10.8%	7.0%	<=0.001 (2)
*Latin America	33.2%	36.3%	0.315 (2)
**Eastern Europe**	35.5%	37.6%	
**Maghreb**	23.0%	22.2%	
**Sub-Saharan**	7.4%	3.0%	
**Others**	0.9%	0.9%	

* Calculated using only the subgroup considered as immigrants.

(1) p value of the t-test (comparison of means).

(2) p value of the chi square independence test.

**Figure 1:**
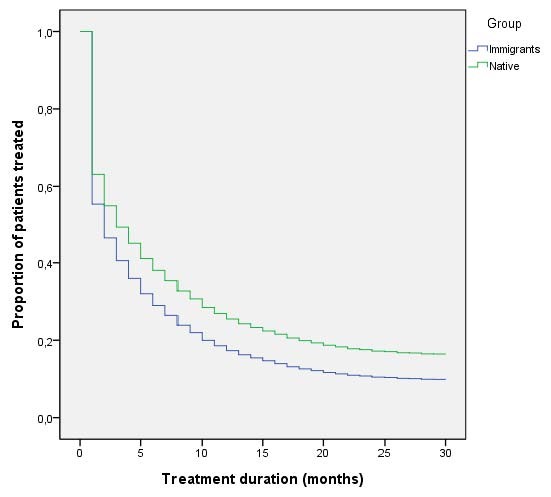
Survival function of treatment duration adjusted for age and sex using a Cox regression model

Treatment duration for each group is shown in Table 3 and the survival functions in Figure 1. The differences between natives and immigrants of all origins were statistically significant.

**Table 3:** Distribution of treatment duration by area of origin

	Median*	**P25**	**P75**	Mean*	95%CI	
**NATIVES**	3	13	1	11.1	10.7	11.5
**IMMIGRANTS**	2	6	1	6.4	5.5	7.4
**Latin America**	2	6	1	7.3	5.5	9.0
**Eastern Europe**	2	6	1	5.8	4.5	7.0
**Maghreb**	2	4	1	6.0	4.0	8.0
**Sub-Saharan**	1	9	1	4.6	1.9	7.3
**Others**	1	4	1	7	0.0	16.9
**TOTAL**	3	12	1	10.7	10.4	11.1

*Parameters calculated with the Kaplan-Meier method.

Half of the immigrants completed two months of treatment, while half of the natives obtained medication for three months (95%CI of the median=2.7-3.3). Of the immigrant groups, the Sub-Saharan Africans and Others presented the lowest median durations (one month).

Excluding the patients who abandoned during the first month, the median duration was nine months (95%CI 8.5-9.5): six months for the immigrants (95%CI 4.9-7) and nine months for the natives (95%CI 8.5-9.5). The differences between natives and immigrants remained statistically significant.

As a whole, the medians of treatment duration were highest in the 35-54years age group; in this age group, median duration was higher in the natives than in the immigrants.

Survival analysis using Cox regression estimated a risk of abandoning/ending treatment in the immigrant group vs. the native group of 1.28 (95%CI=1.16-1.42), adjusted for age and sex.

### Percentage of medication acquired

In all, 76.2% of patients were dispensed at least 80% of the required medication.

Excluding the patients who abandoned during the first month (one package for one month, 100% of required medication dispensed), 59.7% of patients adhered to treatment. No statistically significant differences were found between natives and immigrants with regard to adherence.

### Compliance with treatment

In the whole sample, only 23.8% of patients who began AD treatment remained compliant for a minimum of four months and at the same time acquired more than 80% of the number of packs prescribed (15.3% of immigrants compared with 24.6% of natives, p <0.001).

When we excluded the patients who abandoned during the first month, good compliance increased to 38.2% (29.5% in immigrants and 38.8% in natives). There were no significant differences between the areas of origin of the immigrants.

Table 4 shows the percentage of compliant patients according to sex, age and origin in the group of patients who continued in treatment for a minimum of a month.

**Table 4:** Percentage of compliant patients according to sex, age and origin

	**Compliant patients**	**P**
**Male**	36.5	0.151
**Female**	39.0	
**Age**		
**15-24 years**	24.6	<0.001
**25-34 years**	35.0	
**35-44 years**	39.2	
**45-54 years**	40.9	
**55-64 years**	41.4	
**NATIVE**	38.8	0.005
**IMMIGRANT**	29.5	
**Latin America**	31.8	0.62
**Eastern Europe**	25.0	
**Maghreb**	32.7	
**Sub-Saharan**	42.8	
**Others**	0	

## Discussion

### Main results

Our results show that half of the immigrant patients who begin treatment with AD obtain medication for only two months, compared with a median of three months in the native group. The average risk of abandoning treatment during follow-up is 28% higher in immigrant patients than in the natives, adjusting for age and sex.

Of the patients who continued treatment beyond the first month, only 29.5% of immigrants presented good compliance, compared with 38.8% of natives (withdrawal of above 80% of prescribed medication over at least four months).

These rates fall well below the current recommendations of European and US scientific societies which indicate the initiation of ADT in moderate or severe episodes of depression, and prolongation of treatment during the acute phase (between four and eight weeks) with an additional period of four to nine months to prevent relapses and to recover normal psychosocial functions. Duration may be longer if the clinical history of the patient so requires. Overall, a minimum treatment duration of six months is recommended.

### Comparison with other published studies

 Early abandonment of ADT is a well-documented, persistent occurrence in spite of attempts to encourage adherence. The percentages of abandonment during the first month published in previous studies are similar to ours, with figures ranging between 33% and 42% [1,7-9]. As regards the duration of antidepressant treatment, a study performed with a similar methodology [10] corroborates our data since it obtained a median treatment duration of three months, which the authors attributed to the high percentage of early abandonment. Other percentages of treatment abandonment published range between 50% during the first three months [11], 62.4% at the end of the third month [1] and 44% at six months [12]. An earlier study in our region obtained a discontinuation rate of 56% during the first four months [3].

Much less is known about the pattern of use of ADT by the immigrant population. Most of the data recorded are indirect and come from studies not specifically aimed at this population or studies that do not conduct comparisons with natives. Van Geffen et al. [13] concluded that the risk of not starting treatment or of acquiring only one prescription is higher when the indication is not specific, and that the risk of early abandonment is five times higher in some immigrant groups than in natives. Van Dijk et al. [14] found a relation between the risk of abandonment of ADT and immigrant status, the type of drug prescribed, and comorbidity. Hasen et al. [7] associated early abandonment with foreign citizenship, low educational level, and low income, and Olfson et al. [1] related abandonment in the first 30days with being Hispanic, low educational level, and low family income.

Our study provides evidence that immigrants of all origins present higher percentages of early discontinuation of ADT and lower median treatment durations than the native population in our health region, regardless of their geographic origin. These findings corroborate those of a previous study by our group that showed a lower exposure to ADT in immigrants than in the native population; this tendency was found in all the ethnic groups, but was particularly marked in the sub-Saharans [15].

Although the study was not designed to analyse the causes of the differences between the two populations, the results oblige us to reflect on the factors that may contribute to this situation and to assess whether these differences indicate inequalities in health care.

Clearly, some of the factors associated with poor adherence will affect both immigrants and natives and therefore should be considered in the global strategy for preventing non-compliance in all patients. However, in the immigrant population, in which the economic, social and family situations tend to be less favourable, factors associated with socioeconomic status and environment are likely to predominate. The measures for preventing non-adherence, then, go beyond the jurisdiction of the health system and need to be addressed at the broader level of social and economic policy. Non-adherence due to the cost of medication, a common finding in studies in the US comparing white elderly with their black or Hispanic peers [16], can be included in this group. In Spain the public health system offers pharmaceutical coverage for AD, under which patients pay only 10% of the total cost, a situation that minimizes (but does not eliminate entirely) this effect.

With respect to the factors associated with immigrant status, it might be hypothesized that the shorter duration of ADT and the lower rates of compliance were due in part to a diagnostic bias. Incorrect diagnosis might lead to ineffective prescription and to early drop-out due to the lack of improvement. We cannot test this hypothesis because we do not have information on the diagnoses made. Nonetheless, the difficulties involved in diagnosing psychiatric syndromes in populations from different cultures are well known, due both to the barriers in communication and to the possible unreliability of the standard diagnostic instruments and criteria such as the DSM-IV in these populations.

The beliefs, expectations and attitudes towards mental illness that form part of the cultural background of each ethnic group should also be taken into consideration. They may be very different from those of the host society and may well affect adherence to pharmacological and non-pharmacological treatments.

Most authors agree that patients attitude towards ADT is one of the strongest predictors of adherence. Aikens et al. found that subjects of advanced age, with more severe symptoms, greater acceptance of the biochemical cause of the illness and expectations of persistence of the symptoms were more likely to perceive the need for treatment; these authors also found that patients who had never received AD treatment and who were not certain of the cause of their symptoms were more likely to associate therapy with negative effects [17]. Van Geffen et al. interviewed patients in the early stages of ADT; those who dropped out were less involved in decision-making, had less faith in their doctor, reported a lack of information and presented a negative attitude to ADT, and were not convinced of the necessity of taking it [18]. Reviewing studies of the differences in treatment of depressive and anxiety disorders in ethnic minorities, Schraufnagel et al. detected several obstacles to appropriate treatment, such as the beliefs about treatment mentioned above, stigma, the inability of the doctor to motivate the patient and the biological differences that condition the treatment. Nonetheless, these authors conclude that if the treatment is adequate the results in minorities are comparable to those achieved in the native population [19].

Finally, in relation to these results, it should be recalled that health professionals may lack the intercultural competence necessary to provide optimal treatment to their patients. Their unfamiliarity with their patients culture may present a serious obstacle to early diagnosis and to the development of the therapeutic alliance, both of which are indispensable for obtaining positive results.

All things considered, the cultural identities of immigrant patients and their previous experiences of mental illness are likely to affect their ability to understand, accept and believe in the benefits of treatment. This hurdle appears to be especially significant in certain groups such as the sub-Saharan Africans, the population with the lowest median treatment duration and the lowest overall exposure to ADT reported in the studies mentioned above, and a lower exposure to antipsychotics than the rest of the minorities [20].

These findings have serious implications for the care of immigrant patients: implications for the use and provision of health services (i.e., the need to reach migrant groups considering the cultural specificity of each of them, especially in early stages of treatment of mental illness), implications for health policy (i.e., identifying the factors that limit access to mental health services) and implications for future research (i.e., analysing in depth the causes of the differences observed).

### Limitations

The main limitations of the study are to do with the sample selection. The sample is not representative of the entire immigrant population in the region, only of the immigrants who appear in the municipal census. Furthermore, exposure to ADT is estimated on the basis of medication withdrawn from the pharmacies in the region, with the result that any medication obtained in another health region is not reflected; however, this situation would affect a minimal number of cases in both groups, as the active population recorded in the regional health register remained stable over the period of analysis. The amounts of ADT received over the counter were also considered to be minimal, as prescriptions for this kind of drug are obligatory.

We are aware that our use of the concept of immigrant is excessively general. Although we sought to distinguish between immigrant groups according to their geographical origin, this approach also ignores the existence of differences between countries, between ethnic groups inside the same country and between individuals in the same ethnic group. For this reason, on the basis of these general data it would be interesting to focus on those groups in which the greatest differences were found with respect to the general population.

The lack of diagnoses is a limitation inherent to many studies performed on the basis of claims databases, since this method does not identify the reason for the prescription or the reason for the ending of treatment (i.e., cure or abandonment). This means that the data should be interpreted with care. Similarly, the analysis of drugs dispensed does not indicate the use that patients make of this medication, but reflects the intention to comply with treatment; even so, most authors agree that dispensation is the best measurement of medication use in large populations in habitual clinical practice.

Another limitation is the fact that we were unable to determine primary non-compliance, that is, the number of patients who are prescribed ADT but do not acquire the first pack. This information should be added to the percentage of non-compliance detected, but at the time of the study we did not have the prescription data needed to carry out this estimation.

For the same reason, we were unable to check the dose of ADT prescribed. We assumed that the daily dose prescribed corresponded to the definitions of the WHO Collaborating Centre for Drug Statistics Methodology, and that the prescribing physicians followed the recommendations of this institution.

## Conclusions

In this health region in Spain, half of the immigrant patients who began ADT dropped out during the second month of treatment.

Of the half that continued, only one of every three obtained more than 80% of the packs prescribed in a minimum period of four months, in spite of the current recommendations in favour of longer treatments. Some immigrant groups such as sub-Saharan Africans present even shorter durations of treatment. This situation seems to reflect an inadequate provision of mental health care in this population.

Few studies of the prevalence of mental illness and adherence to ADT have been performed in immigrant groups in our environment. Since the objective of the Spanish national health system is to guarantee an equitable provision of care, it is vital to attend to the needs of the immigrant population and to ensure adequate access and use of health services. To achieve this aim, we need valid data from studies that are specifically designed to detect immigrants needs and propose appropriate responses. These studies should not only provide quantitative data but should also include qualitative methodologies to explore the immigrant perspective.

## Pre-publication history

The pre-publication history for this paper can be accessed here:

http://www.biomedcentral.com/1471-2458/12/256/prepub
